# Guillain-Barré Syndrome as a Presenting Symptom in Breast Cancer: The Importance of Considering Paraneoplastic Neurologic Syndrome

**DOI:** 10.7759/cureus.17932

**Published:** 2021-09-13

**Authors:** Samragnyi Madala, Kira Macdougall, Gerard Morvillo, Robert Guarino, Alisa Sokoloff

**Affiliations:** 1 Internal Medicine, Hofstra Northwell School of Medicine, Staten Island, USA; 2 Internal Medicine, Northwell Health, New York, USA; 3 Pathology, Northwell Health, New York, USA; 4 Hematology/Oncology, Northwell Health, New York, USA

**Keywords:** malignancy, chemotherapy, breast cancer, paraneoplastic neurologic syndrome, guillain-barre syndrome

## Abstract

Paraneoplastic neurological syndromes (PNS) are a group of rare immune-mediated disorders with neurological sequela in cancer patients. It usually occurs when an immune response against a systemic tumor is incorrectly directed to the nervous system. Compared to other reported manifestations of PNS in breast cancer, Guillain Barre syndrome (GBS) is exceedingly rare. There is only one other reported case in the literature of GBS that was diagnosed in a breast cancer patient. We report the second recorded case of a 61-year-old female with a history of early-stage breast cancer, who presented with symptoms of lower extremity weakness initially suspected to be GBS but later found to have been recurrent breast cancer. No specific guidelines are available for the treatment of PNS. Treatment of underlying malignancy with chemotherapy and immunotherapies are usually recommended.

## Introduction

Paraneoplastic neurological syndromes (PNS) are a group of rare immune-mediated disorders with neurological sequela in cancer patients. It usually occurs when an immune response against a systemic tumor is incorrectly directed to the nervous system [1]. The incidence of these syndromes is approximately one in 300 of all patients with malignancy [2]. It is most commonly seen in patients with lung, ovarian, lymphatic systems, and breast malignancies. PNS has been reported in breast cancer as early as 1968 [3]. Neurological symptoms in PNS include gait disturbance, dysphagia, decreased muscle tone, decreased fine motor coordination, decreased speech intelligibility, memory loss, visual problems, sensory deficits, dizziness, and epileptic seizures [4,5]. We report the case of a 61-year-old female with a history of early-stage breast cancer, who presented with symptoms of lower extremity weakness initially suspected to be Guillain Barre syndrome (GBS). Only later was she diagnosed with PNS secondary to breast cancer.

## Case presentation

**Figure 1:**
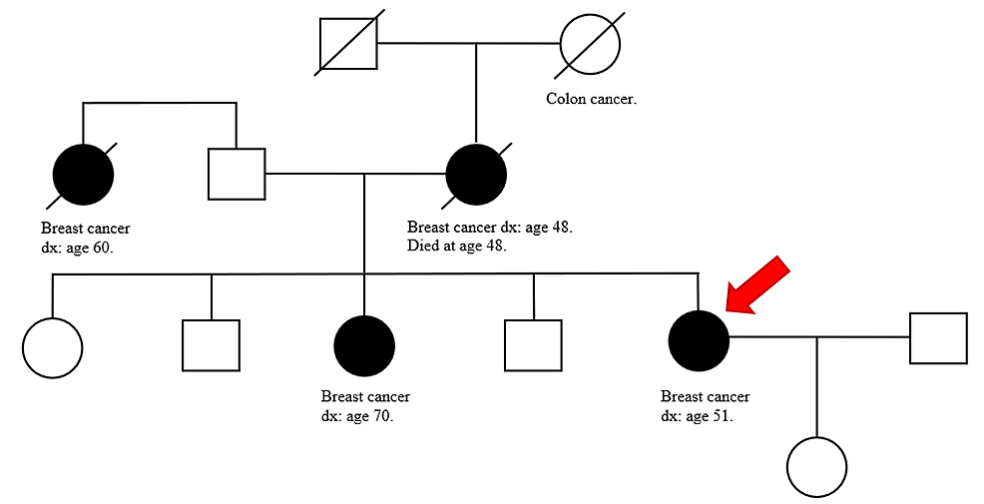
Family pedigree illustrating the age of relatives when they were diagnosed with breast cancer. Black symbols represent family members that were diagnosed with breast cancer. The red arrow indicates our patient. dx = diagnosis

**Figure 2:**
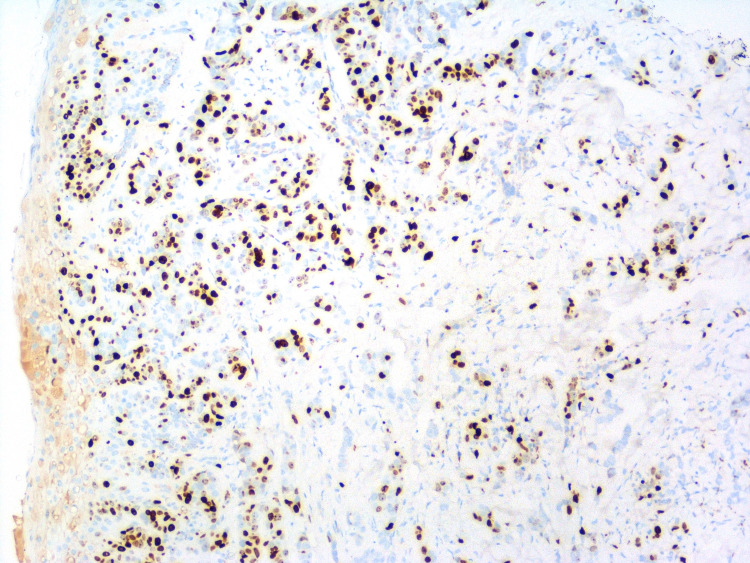
Malignant ductal epithelial cells with Ki-67 proliferative index showing 50% positive stained cells at 100x total magnification.

**Figure 3:**
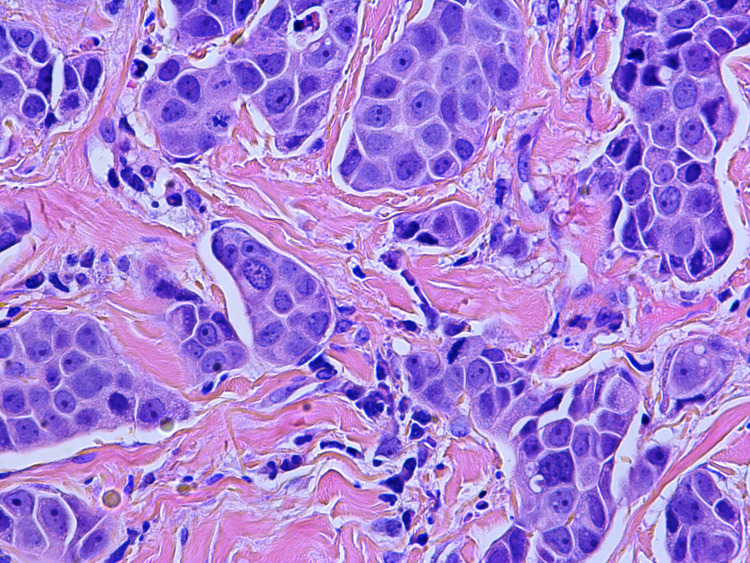
Hematoxylin and eosin (H&E) slide of Invasive poorly differentiated ductal carcinoma confined to dermis with focal lobular features at 400x total magnification

**Figure 4:**
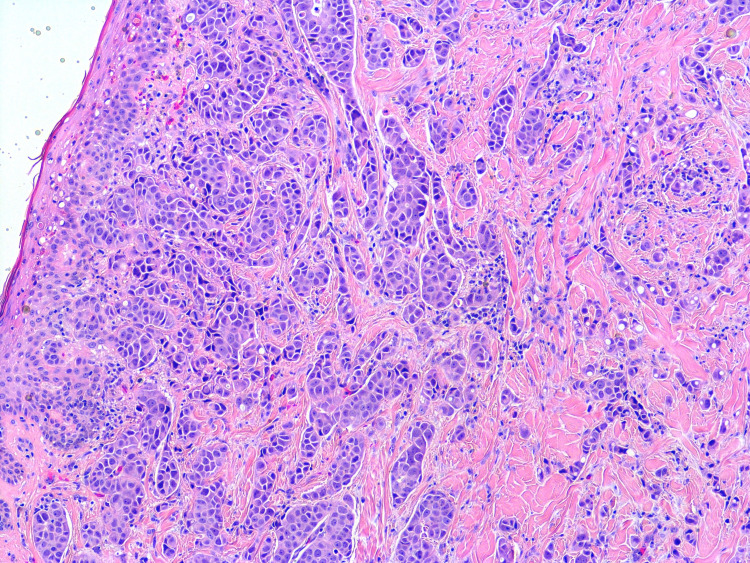
Hematoxylin and eosin slide of Invasive poorly differentiated ductal carcinoma confined to dermis with focal lobular features at 100x total magnification

**Figure 5:**
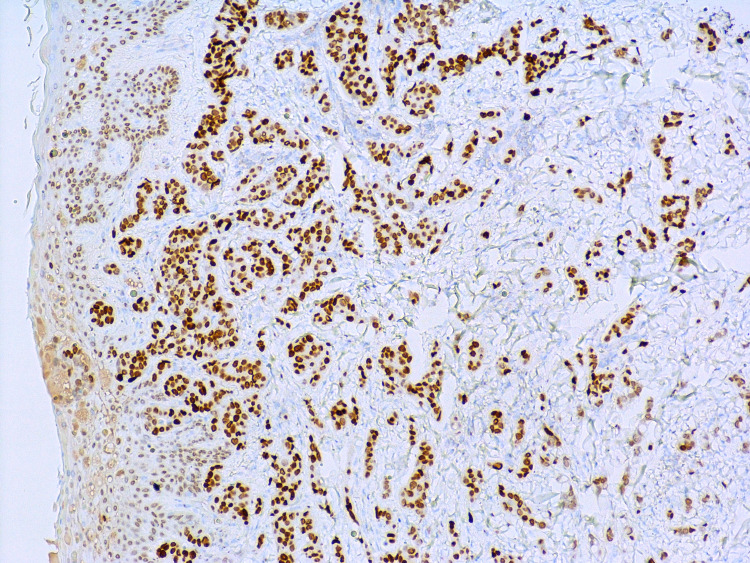
Malignant ductal epithelial cells staining positive with GATA-3 nuclear stain at 100x total magnification

A 61-year-old female with a past medical history of Stage IB breast cancer s/p right-sided mastectomy ten years prior to presentation, hypertension, diabetes mellitus type 2, GBS (two months ago) on treatment with monthly outpatient intravenous immunoglobulin (IVIG) was presented to the emergency department from her nursing home with a chief complaint of inflammation in her left breast. While at the nursing home, erythema of the left breast along with axilla was noticed for which she was treated with a course of cephalexin and clindamycin for possible cellulitis. However, symptoms worsened, and she presented to the hospital. On examination vital signs were stable and breast examination was remarkable for erythema and swelling of the left breast and axilla along with palpable left axillary lymph nodes. Oncology service was consulted for inflammatory changes of the left breast and draining axillary lymph nodes. Her oncological history was reviewed for breast cancer s/p right-sided mastectomy (ten years prior) with pathology revealing infiltrating ductal carcinoma, moderately differentiated. The patient did not receive radiation or chemotherapy. Three years after mastectomy, the patient stopped following up with her oncologist. Her last mammogram was four years ago. Patient reports being negative for the BReast CAncer (BRCA) gene. She has a strong family history of breast cancer. Breast cancer in her mother was diagnosed at the age of 48 years, her sister was diagnosed at the age of 70 years, and her paternal aunt was diagnosed at the age of 60 years (Figure 1). Punch biopsy of the left 4 mm peri areolar mass was obtained for the new breast lesion. Metastatic workup with computerized tomography (CT) scan of chest/abdomen/pelvis showed left axillary lymphadenopathy with largest one measuring 1.7 cm, left internal mammary lymph node (LN) 6 mm, shoddy paratracheal and subcarinal lymph nodes. Bone scan and CT scan of the brain did not show evidence of metastasis. Pathology showed non-ulcerated areolar/periareolar skin with invasive poorly differentiated ductal carcinoma confined to the dermis with focal lobular features and single-cell necrosis (Figures 2-5). Immunohistochemical staining of breast tissue was negative for estrogen receptor (ER), progesterone receptor (PR), and human epidermal growth factor receptor2 (HER2) results thereby classifying the patient as having stage IIIC pathologic stage group. Breast Cancer 15.3 was elevated to 48.4. The patient was also found to have positive GATA3, mammaglobin, gross cystic disease fluid protein 15, cytokeratin (CK) 7, E-cadherin. Negative for CK 20, S-100. Positron emission tomography-computed tomography (PET/CT) showed no evidence of active biologic tumor activity, specifically no F-fluorodeoxyglucose (FDG) avid thoracic lymphadenopathy.

The patient’s lower extremity weakness was still present and was attributed to being secondary to the paraneoplastic syndrome. A paraneoplastic neurological panel was sent for antibodies including anti-RI, anti-Yo, and anti-hu as noted in Table 1. Cerebrospinal studies showed albumin cytologic dissociation. Her previous treatment with IVIG had minimal improvement and the last dose was prior to admission. The patient’s symptoms initially responded to IVIG treatment but with recurrent symptoms days after treatment. Patient was started on neoadjuvant chemotherapy which showed improvement in her lower extremity weakness. Few months after chemotherapy, left radical mastectomy with axillary lymph node dissection was performed followed by chest wall irradiation. Final surgical pathology revealed 20 mm invasive poorly differentiated ductal carcinoma with metastatic carcinoma present in four axillary lymph nodes. Immunohistochemistry showed ER, PR positive, HER2 negative with cancer being staged as ypT1C, ypN2a, ypM0. Chemotherapy improved weakness in her extremities and she was discharged back to a nursing home with subsequent follow-up with Oncology for further treatment of her breast cancer.

**Table 1:** Table showing antibodies panel sent for Paraneoplastic neurological syndrome

Antibodies:	Results (Normal Range)
Voltage gated ca channels	28.7pmol/L (0.0-24.5pmol/L)
Asialo-GM1 antibodies	negative
IgG/IgM antibodies for: GM1, GM2, GD1a, GD 1b, GQ1b	negative
Neuronal nuclear Ri antibody, western blot	negative
Sulfatide antibodies	negative
Anti Yo antibodies	positive
Anti HU antibodies	negative
Anti-Musk antibodies	negative
Acetylcholinesterase	4290 (2504-6297 IU/L)
Cancer antigen, breast Ca 15.3	48.4 (<25 U/mL)

## Discussion

According to the 2020 global cancer burden estimates released by the International Agency for Research on Cancer (IARC), female breast cancer is now the leading cause of cancer incidence worldwide. Out of the 19.3 million new cancer cases in 2020, 2.3 million (11.7%) were diagnosed with breast cancer. It ranks fifth in cancer mortality worldwide with an estimated 685,000 (6.9%) deaths [6]. Though several advancements have been made in the diagnosis through screening initiatives, breast cancer may still present as a paraneoplastic neurologic manifestation. As our patient, the presentation is often misleading and difficult to diagnose, which may lead to a delay in cancer diagnosis.

PNS was previously divided into definite or possible, classical, or non-classical types. The classification was made based on the presence or absence of tumor, time from tumor diagnosis, presence of onconeural antibodies, and response to therapy [7]. Several antibodies can be used as biomarkers to aid in the diagnosis of PNS. These antibodies are commonly named onconeural antibodies and can affect either the central, peripheral, or autonomic nervous systems. Anti-neuronal nuclear type antibody (Anti-Hu), Purkinje cell antibody type -1 (Anti-Yo), Collapsin response-mediator protein-5 (Anti-CV2), Anti-neuronal nuclear antibody type-2 (Anti-Ri), Anti-Ma2, Anti-amphiphysin are some of the well-characterized onconeural antibodies [7]. The presence of onconeural antibodies aids with the diagnosis when present, but their absence cannot exclude the possibility of a paraneoplastic neurologic syndrome [3]. Approximately two-thirds of the patients with PNS secondary to breast cancer are known to have positive antibodies [3].

There have been many recognizable patterns in the presentation of PNS in the last few decades. In 2019, an updated scoring system was developed to facilitate better diagnosis of PNS, which divides the neurological presentations as high-risk and intermediate-risk phenotypes. High-risk neurological phenotypes include encephalomyelitis, limbic encephalitis, rapidly progressive cerebellar syndrome, opsoclonus-myoclonus, sensory neuropathy, gastrointestinal pseudo-obstruction (enteric neuropathy), Lambert-Eaton myasthenic syndrome. Intermediate risk phenotypes include limbic encephalitis, anti-N-methyl-D-aspartate receptor encephalitis, Morvan syndrome. Where high-risk phenotypes are mostly triggered by cancer and need a workup for an underlying malignancy, intermediate risk phenotypes can be seen with or without cancer [8]. GBS associated with breast cancer is included under intermediate/non-classical PNS [7].

Compared to other reported manifestations of PNS in breast cancer, GBS is exceedingly rare. There is only one other reported case in the literature of GBS that was diagnosed in a breast cancer patient [9]. In this case, the patient presented with weakness and bloody nipple discharge [9].

There is evidence in recent decades on the immunogenicity of breast tumors. The presence of tumor-infiltrating lymphocytes (TILs) has been observed as a good prognostic indicator particularly in triple-negative breast cancer [10]. In this era of immune checkpoint inhibitors (ICI), there is a possibility of developing autoimmune neurologic disorders after treatment with ICIs. There is evidence of developing neurological syndromes associated with anti-Ma2 encephalitis [11] and Hu antibodies after starting ICIs making it harder to differentiate if the symptoms are related to PNS versus treatment with ICI.

No specific guidelines are available for the treatment of PNS. Treatment of underlying malignancy with chemotherapy and immunotherapies is usually recommended. Common first-line immunotherapies are intravenous methylprednisolone, IVIG, and plasma exchange. Treatment must be initiated as early as possible to prevent irreversible neuronal loss [12].

## Conclusions

We present the case of a patient who was diagnosed with breast cancer a few months after being diagnosed with GBS. She tested positive for Anti-Yo antibodies. Regardless of the presence or absence of antibodies, it is important to maintain a high suspicion for PNS from underlying malignancy when a patient presents with symptoms of GBS, as to not miss this important diagnosis.

## References

[1] Ruiz-García R, Martínez-Hernández E, Saiz A, Dalmau J, Graus F (2020). The diagnostic value of onconeural antibodies depends on how they are tested. Front Immunol.

[2] Vogrig A, Gigli GL, Segatti S (2020). Epidemiology of paraneoplastic neurological syndromes: a population-based study. J Neurol.

[3] Fanous I, Dillon P (2015). Paraneoplastic neurological complications of breast cancer. Exp Hematol Oncol.

[4] Minisini AM, Pauletto G, Bergonzi P, Fasola G (2007). Paraneoplastic neurological syndromes and breast cancer. Regression of paraneoplastic neurological sensorimotor neuropathy in a patient with metastatic breast cancer treated with capecitabine: a case study and mini-review of the literature. Breast Cancer Res Treat.

[5] (2021). Paraneoplastic syndromes information page. https://www.ninds.nih.gov/disorders/all-disorders/paraneoplastic-syndromes-information-page.

[6] Sung H, Ferlay J, Siegel RL, Laversanne M, Soerjomataram I, Jemal A, Bray F (2021). Global Cancer Statistics 2020: GLOBOCAN Estimates of Incidence and Mortality Worldwide for 36 Cancers in 185 Countries. CA Cancer J Clin.

[7] Graus F, Delattre JY, Antoine JC (2004). Recommended diagnostic criteria for paraneoplastic neurological syndromes. J Neurol Neurosurg Psychiatry.

[8] Graus F, Vogrig A, Muñiz-Castrillo S (2021). Updated diagnostic criteria for paraneoplastic neurologic syndromes. Neurol Neuroimmunol Neuroinflamm.

[9] Tuna F, Tafitek E, Ozdem H, Duzce E, Tuna H (2016). Breast cancer in a woman with Guillain-Barre syndrome: A reminder to consider paraneoplastic neurological syndrome. Turkish Journal of Geriatrics.

[10] Criscitiello C, Esposito A, Gelao L (2014). Immune approaches to the treatment of breast cancer, around the corner?. Breast Cancer Res.

[11] Vogrig A, Fouret M, Joubert B (2019). Increased frequency of anti-Ma2 encephalitis associated with immune checkpoint inhibitors. Neurol Neuroimmunol Neuroinflamm.

[12] Devine MF, Kothapalli N, Elkhooly M, Dubey D (2021). Paraneoplastic neurological syndromes: clinical presentations and management. Ther Adv Neurol Disord.

